# Treatment of anaerobically digested pig manure by applying membrane processes for nutrient recovery and antibiotics removal

**DOI:** 10.1007/s11356-024-33313-x

**Published:** 2024-04-13

**Authors:** Vera Proskynitopoulou, Anastasios Vourros, Ioannis Garagounis, Panagiotis Dimopoulos Toursidis, Souzana Lorentzou, Panagiotis Kougias, Anastasios Zouboulis, Kyriakos D. Panopoulos

**Affiliations:** 1 ARTEMIS Laboratory, Chemical Process and Energy Resources Institute, Centre for Research & Technology Hellas, 57001 Thessaloniki, Greece; 2 Chemical and Environmental Technology Laboratory, Department of Chemistry, Aristotle University of Thessaloniki, 54124 Thessaloniki, Greece; 3 Hellenic Agricultural Organisation-DEMETER, Soil and Water Resources Institute, 57001 Thessaloniki, Greece

**Keywords:** Digestate processing, Membrane treatment, Nutrient recovery, Antibiotics presence, Circular economy, Selective electrodialysis, Fertilizers

## Abstract

A combination of membrane processes was applied to treat the digestate produced after the anaerobic treatment of pig manure in a biogas plant, aiming towards the recovery of nutrients and effective water treatment for potential reuse. Initially, coarse filtration (sieving and microfiltration) was used to remove particles larger than 1 µm, followed by ultrafiltration, to reduce the suspended solids concentrations below 1 g/L. Subsequently, selective electrodialysis is employed to recover the main nutrient ions, primarily ammonium and potassium. The ion-depleted digestate is then fed to a reverse osmosis unit, where clean water was recovered, yielding a by-product (concentrate) stream enriched in phosphates and organics content. The presence of antibiotics and the concentrations of heavy metals were monitored during all treatment stages to assess their behavior/removal in the various membrane processes. The results indicate that almost 51% of the digestate could be recovered as water free from ions and antibiotics, suitable for reuse in the biogas plant for process needs and irrigation purposes. The selective electrodialysis process can recover 51% of initial NH_4_^+^ content (corresponding to 96% of the electrodialysis feed), while the remainder largely ended up in the ultrafiltration concentrate. A similar behavior was observed for the case of K^+^, while approximately 68% of the phosphates content was retained by the coarse filtration process, with another 24% remaining in the ultrafiltration concentrate and the remaining 8% in the reverse osmosis concentrate. Most of the antibiotics and heavy metals were retained by the coarse and ultrafiltration steps, with smaller amounts detected in the reverse osmosis concentrate.

**Supplementary Information:**

The online version contains supplementary material available at 10.1007/s11356-024-33313-x.

## Introduction

During the last decades, the shift towards a circular bio/economy and environmentally friendly waste management has increasingly been considered as mandatory (Intergovernmental Panel on Climate Change 2023). The recovery of nutrients from wastes and their reuse is an alternative and ecologically-sound approach to partially replace the use of chemical fertilizers, which are associated with energy-intensive production methods and higher greenhouse gas (GHG) emissions. This replacement is associated with better wastewater purification, aiming to protect surface and ground waters (Chojnacka et al. 2020).

Anaerobic digestion is considered by several researchers (e.g., Bdiri et al. 2020; Khalid et al. 2011; Kiyasudeen et al. 2016) to be the cornerstone of sustainable waste management, converting various organic wastes, such as animal manure, into biogas (considered a renewable energy source) that can be used, e.g., in electricity production. The main by-product of the anaerobic digestion is digestate, which is widely used to improve the quality of agricultural soils with inorganic and organic components (Nkoa 2014). However, fluctuations in the raw materials used as feedstocks in anaerobic digesters/bioreactors of biogas plants usually result in the production of digestates with varying chemical composition (Akhiar 2017; Tambone et al. 2017; Teglia et al. 2011). Furthermore, it has been previously reported that digestates originating from pig manure treatment may contain several problematic impurities, including antibiotics and heavy metals (Gros et al. 2019; Wolters et al. 2016; F. Zhang et al. 2012). This issue can create significant secondary issues, such as challenges in further utilization and, consequently, major management issues for storage or disposal. If not managed properly, the direct disposal of digestate onto the agricultural soil may result in the uncontrolled leaching of nutrients and other undesirable constituents, potentially contaminating soil, as well as surface and ground waters (Huang et al. 2017).

Currently, the most popular management strategy is to separate the digestate into liquid and solid fractions, reducing its volume and hence, the respective transport costs. Screw separation, centrifugation, and filter presses are the main methods commonly used for the digestate separation, although these processes are rather energy-intensive and not particularly efficient for achieving solid/liquid separation. Alternatively, the use of membrane separation processes and ammonia stripping for nitrogen recovery have been suggested for treating the liquid fraction (Al Seadi and Lukehurst 2012; Logan and Visvanathan 2019). Furthermore, although other cutting-edge technologies have been explored recently, such as electrodialysis, membrane distillation, and microbial cell recovery, they have not been used yet on a larger scale (Lech et al. 2023; Z. Zhang et al. 2020). On the other hand, composting and drying are considered the two most commonly applied methods for managing the solid fraction of digestate, either naturally or through the application of external heat (Guilayn et al. 2020).

Recent innovations in membrane technology fields are attracting serious attention, making them generally preferred over other alternatively utilized approaches due to their minimal consumption of chemical reagents and potential cost-effectiveness. As a result, they can play a crucial role for the treatment of waste from numerous industrial areas, including food production, chemical synthesis, as well as water and wastewater treatment (Daufin et al. 2001; Ezugbe and Rathilal 2020). The ability of pressure-driven membrane processes, including ultrafiltration, nanofiltration, and reverse osmosis, to separate and concentrate nitrogen or phosphorus from feed solutions, creating a product/solution with higher fertilizer value has been extensively studied for the treatment of digestates (Adam et al. 2018; Gienau et al. 2018; Vaneeckhaute et al. 2012; Waeger et al. 2010; Zacharof et al. 2019).

Nevertheless, the current study seeks to develop an integrated technological approach for processing digestate within a more compact framework, aiming to reduce the overall volume of digestate, recover nutrients from the digestate’s liquid fraction, remove antibiotics, and produce clean water. Pig manure anaerobically digested in a decentralized anaerobic digestion (biogas) plant is processed by sequentially applying several membrane technologies, including microfiltration and ultrafiltration (mainly for the removal of solids and organic matter), selective electrodialysis as an alternative approach for recovering nutrients ions, and reverse osmosis for further antibiotic removal and clean water production.

## Experimental

### Biogas plant digestate

In the performed experiments, the liquid fraction of a pig manure anaerobically produced digestate was used. It was obtained from a small biogas plant (with 100 kWe output) located on a pig farm outside the town of Servia, Kozani, Greece. The manure was washed from the pigpens to the anaerobic digester downhill of the farm, with the help of gravity, while an amount of corn silage was also added with a weight ratio of 1:10 of the manure slurry. This leads to a digestate with more than 97% water content. The digestate subsequently passed through a screw press, and the liquid fraction is stored in an aerated lagoon for further use by local farmers, whereas the concentrated solids fraction is also distributed to local farmers.

### Digestate processing equipment

**Fig. 1:**
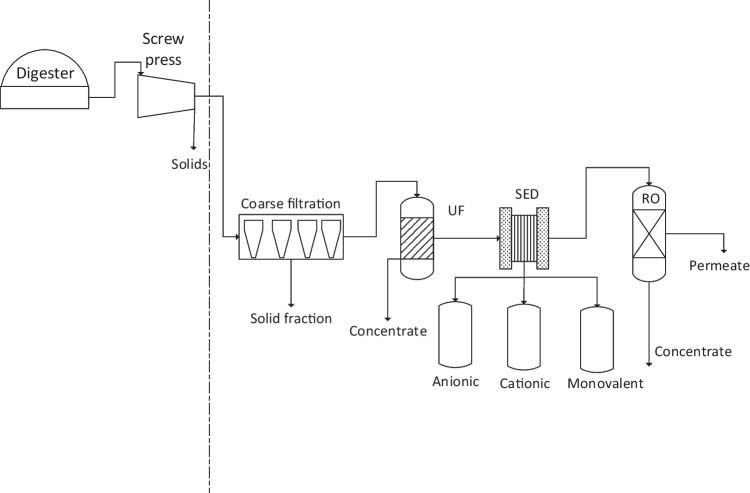
Schematic diagram of the employed membrane treatment process for the anaerobically produced manure digestate

The current combined process aims to recover nutrients, clean water, and solids, for use as soil amenders, from the digestate. To achieve these targets, it consists of sieving, microfiltration (MF), and ultrafiltration (UF) units to remove most of the suspended solids, followed by the selective electrodialysis (SED) unit to recover the nutrient ions from the pre-filtered digestate, and finally, the reverse osmosis (RO) unit to produce water (Fig. 1).

To reduce the initial solids loading and make it more manageable for the subsequent bag filters (MF unit), the digestate was first sieved through a 0.5-mm sieve. The MF unit consisted of four polyester bag filters arranged in cascade mode. These 4 filters were placed in 20″ filter housings and in this set of experiments aimed to progressively separate particles down to 100, 50, 25, and 1 µm. The unit was also equipped with a centrifugal pump capable of flow rates up to 1200 L/h, used to transfer the digestate from a 1000-L storage tank, through the filters and into the next storage tank, holding the separated liquid fraction after the MF application, also being used as feed for the following UF unit.

The applied UF unit was a dead-end system (obtained from SolarSpring GmbH, Germany), consisting of two parallel tubes fitted with hollow fiber membranes (made of PESM) with a 0.9 mm bore, 20 nm pore size, and an effective area of 6 m^2^ each. The unit also comprises a 5-L backflush tank, which uses some of the permeate to partially clean the membranes by automatically reversing the flow of liquid at preset time intervals. The UF feed came from the post-MF storage tank with the help of a reciprocal pump. The transmembrane pressure started at 0.9 bar and was allowed to increase up to 5 bars (maximum), as the fouling (flow resistance) of membranes gradually increased, which was flushed (cleaned) intermediately by the backflush tank content.

The SED unit (obtained from PCCell GmbH, Germany) focused on a 25 cm × 25 cm cell stack (ED Q380), containing twenty cells. Each cell consisted of standard anionic and cationic ion exchange membranes, as well as monovalent anionic and monovalent cationic ones, arranged to produce three product streams, kept in three separate 25-L tanks. The membrane arrangement to achieve selective ion separation was described in a previous publication by the authors (Proskynitopoulou et al. 2024). The anionic product was kept in tank B, the cationic in tank D, and the monovalent, also referred to as brine, in tank C. The feed tank (tank A) was filled with 20 L of UF permeate, while the product tanks were filled with 10 L of 1% NaCl solution. The purpose of NaCl is to help the balance of electrical charges in the tanks as the nutrient ions move in or out and to increase the initial conductivity of product streams, expediting the transfer of ions from the digestate. A fifth tank containing a 0.25 M Na_2_SO_4_ solution was used as feed for the electrodes’ compartments, insulating them from the process ions. These streams fed through the cell at a flow rate of 250 L/h. The cell operating voltage was kept at 30 V. The duration of each SED run was determined by the conductivity of digestate; runs terminated when the measured conductivity value dropped below 0.8 mS/cm. In order to produce more concentrated streams, five consecutive feed batches were processed with the same product solutions.

The RO unit was a high throughput system (obtained from SPECTRUM, UK) with four membranes running in parallel. These were spiral-wound polyamide membranes (SRO-4040–2500-LE) with a combined active area of 8.36 m^2^, capable of withstanding feeds with up to 2000 mg/L dissolved solids load or pressures up of about 12 bars. This unit required also a feed pump, which brought the ion-depleted digestate collected from the previous SED unit (at a pressure of about 8 bars) to the unit’s own pump, which further increased the pressure to the respective necessary operating value for the RO system.

Figure 1 shows the overall separation process as examined in this study. The digestate is received directly from the biogas (anaerobic digestion) plant after the initial solid–liquid separation, applied by a typical screw press. For each experiment, 1000 L of digestate was used. The first two units (screw press and coarse filtration unit) are mainly applied for the reduction of the solids load. The coarse filtration unit consists of the sieving and the MF systems. Following this, the application of UF removes practically all solids larger than 0.02 µm, followed by the recovery of nutrient ions in the SED unit, which has 3 product tanks due to its ability to separate the content of ions into three fractions, i.e., anions, cations, and monovalent ions. This ability is considered crucial in order to subsequently obtain valuable fertilizer salts. At the end of this process, the RO unit is used to produce clean water.

### Sampling and analyses

Samples were received from all streams of the integrated process, including the raw digestate, to monitor the operation and conduct the respective mass balances. The samples were stored in PP bottles of appropriate size at 4 °C and analyzed within a week.

The analytical determinations of various parameters for the collected samples are listed in the following.

For the pH measurements, the Bench meter AG 744 (from Metrohm, Switzerland) was used, and for the electric conductivity (EC), the Portable multimeter Multi 3510 IDS (from WTW, Germany) was employed. Total solids (TS) and total suspended solids (TSS) were measured according to 2540C and APHA 2540-D standard methods, respectively. Phosphorus was analyzed as PO_4_-P, using the vanadomolybdo-phosphoric acid colorimetric method, and the absorption was monitored using spectrophotometry (Spectroquant Pharo 300 at 470 nm). Total organic carbon determination was performed according to the APHA 5310 B method using the TOC-L Analyzer (Shimadzu, Japan). BOD_5_ measurement was carried out according to the APHA 5210B method. All aforementioned analyses were conducted in accordance with the Standard Methods for the Examination of Water and Wastewater (APHA 2012).

A Prominence ion chromatograph (obtained from Shimadzu, Japan) was employed for the analyses of cations (NH_4_^+^, K^+^, Ca^2+^, Mg^2+^) using the IC SI-52 4E column (from Shodex, Japan) with a methanesulfonic acid concentration of 4.0 mM at a constant flow rate 1 mL/min, whereas the determination of anions was carried out using the IC SI-52 4E column (Shodex, Japan), with a sodium carbonate concentration of 3.6 mM at a constant flow rate 0.8 mL/min.

The concentrations of heavy metals Cd, Cr, Hg, Ni, Cu, Pb, Zn, and As were determined by the application of inductively coupled plasma-mass spectrometry (ICP-MS, Agilent 7850, USA), following EPA methods 3051A and 6020A (EPA 2007a, b). Prior to analysis, the samples underwent microwave-assisted acid digestion using an Ethos Up digester (obtained from Milestone, Italy).

The analytical determination of antibiotics analysis was performed using the QExactive™ Focus Orbitrap LC–MS/MS system (obtained from Thermo Fisher Scientific, Bremen, Germany), equipped with a heated electrospray ionization source (H-ESI II), while an Ultimate 3000 Ultra-High-Performance liquid chromatography system, comprised from a binary gradient UHPLC system of pumps, a temperature-controlled autosampler, and a column compartment, was used for the respective separation purposes, with the aid of a Thermo Hypersil GOLD aQ column (50 mm × 2.1 mm, 1.9 µm particle size). In all samples, the preliminary solid phase extraction (SPE) was performed by using the Oasis HLB cartridges (200 mg, 6 mL).

The elemental rejection (*R*_*i*_) was calculated according to the following equation, where *C* is the concentration and *f* and *p* indicate feed and permeate, respectively (Gerardo et al. 2015).

$${R}_{i} (\mathrm{\%})=(1-\frac{{C}_{p}}{{C}_{F}})\times 100$$

## Results and discussion

### Digestate characterization

Table 1 shows the results from selected intakes from different feed batches received during the tests involving the treatment of pig manure digestate. The pH ranges between 7.85 and 8.10, with the average conductivity just below 15.00 mS/cm. Considerable variation can be observed in most of the nutrient concentrations, with phosphates varying from 34.50 to 91.90 mg/L, chlorides from 876.00 to 2127.00 mg/L, and total organic carbon from 1.00 to 6.40 g/L. Conversely, potassium and magnesium data vary by less than 25%, ranging from 1308.00 to 1600.00 mg/L and 201.00 to 267.00 mg/L, respectively, while ammonium data exhibit even lower variation (approximately 10%, i.e., from 1998.00 to 2159.00 mg/L).

**Table 1:** Characterization of pig manure digestate used as feed for the examined membrane treatment processes; batches selected to illustrate the variability of different feed streams

Batch	1	2	3
pH	8.04	7.85	8.10
EC (mS/cm)	14.56	15.07	14.69
TS (g/L)	15.45	17.60	13.90
TOC (mg/L)	1075.00	6400.00	4900.00
Na^+^ (mg/L)	493.50	401.80	401.90
NH_4_^+^ (mg/L)	2159.20	2034.00	1998.00
K^+^ (mg/L)	1308.20	1600.00	1600.00
Mg^2+^ (mg/L)	201.00	267.10	219.40
Ca^2+^ (mg/L)	336.40	640.00	470.00
Cl^−^ (mg/L)	878.30	1099.00	2127.00
NO_3_^−^ (mg/L)	186.00	98.80	147.90
PO_4_^3−^ (mg/L)	91.90	40.90	34.50

### Digestate mass flow balance

**Fig. 2:**
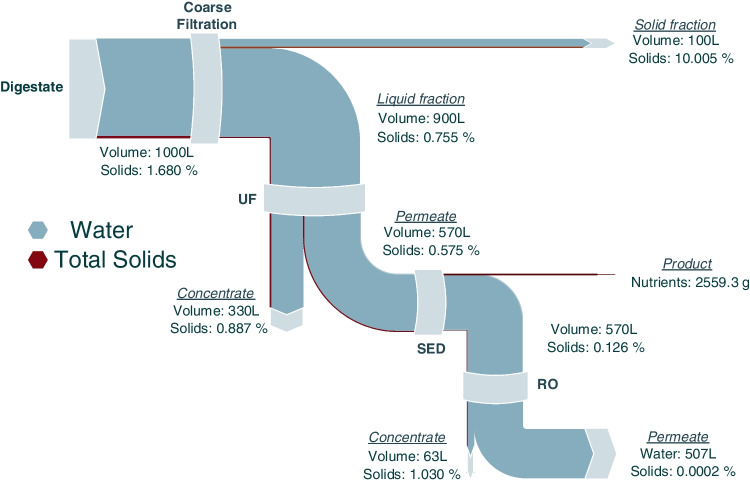
Detailed water and solids mass flow balances through the integrated membrane treatment process of digestate

Figure 2 illustrates the flow balances of water and solids through the integrated process, beginning with the liquid fraction, separated after the screw press. This figure shows that approximately half of the inlet volume was recovered as clean water after the RO application. The digestate, after treatment with SED, was fed to the RO unit, and the concentrate was returned to the feed tank until the highest permitted TDS for this treatment sub-unit was reached, i.e., 2000 mg/L. This approach maximizes the volume of clean water, allowing just over 50% of initial digestate volume to be retrieved as purified water. Specifically, the permeate was 507 L (compared to the 1000 L of digestate), while the produced concentrate was 63 L.

The largest “loss” of water was observed in the UF unit, as depicted in Fig. 2, where 330 L of water followed the solids into the concentrate of this membrane filtration process. This volume is significantly greater than the 100 L “lost” during the coarse filtration preliminary treatment step (i.e., the sieving and MF processes), which removed particles larger than 1 µm. The removal of larger-size particles renders the resulting flow more suitable for the subsequent UF application, enabling this unit to run more smoothly and presenting lower fouling problems.

In combination, the coarse filtration and the UF units removed almost 66% of the initial total solids concentration and nearly all suspended particles, with the majority of the remaining solids being in the dissolved state. Specifically, coarse filtration removed 55% of total solids, reducing their concentration from 16.80 to 7.55 g/L in the permeate stream, which was further treated with UF. In this membrane stage, another 10% of the total solids load was removed, bringing the concentration down to 5.75 g/L. After this stage, 95% of the remaining solids in permeate were in the dissolved stage, while 97.3% of suspended solids had been removed by the three aforementioned filtration steps. This is crucial for mitigating fouling in SED membranes, which are applied in the following treatment step.

After these filtration steps, the digestate was directed to the SED unit for the subsequent separation of nutrient ions. The SED removes only the charged species (ionic forms) from the digestate, and as a result, practically no water was removed with these (dissolved) solids. The ion-free SED effluent was subsequently treated by applying RO, reducing the solids concentration in final permeate down to 0.002 g/L.

**Fig. 3:**
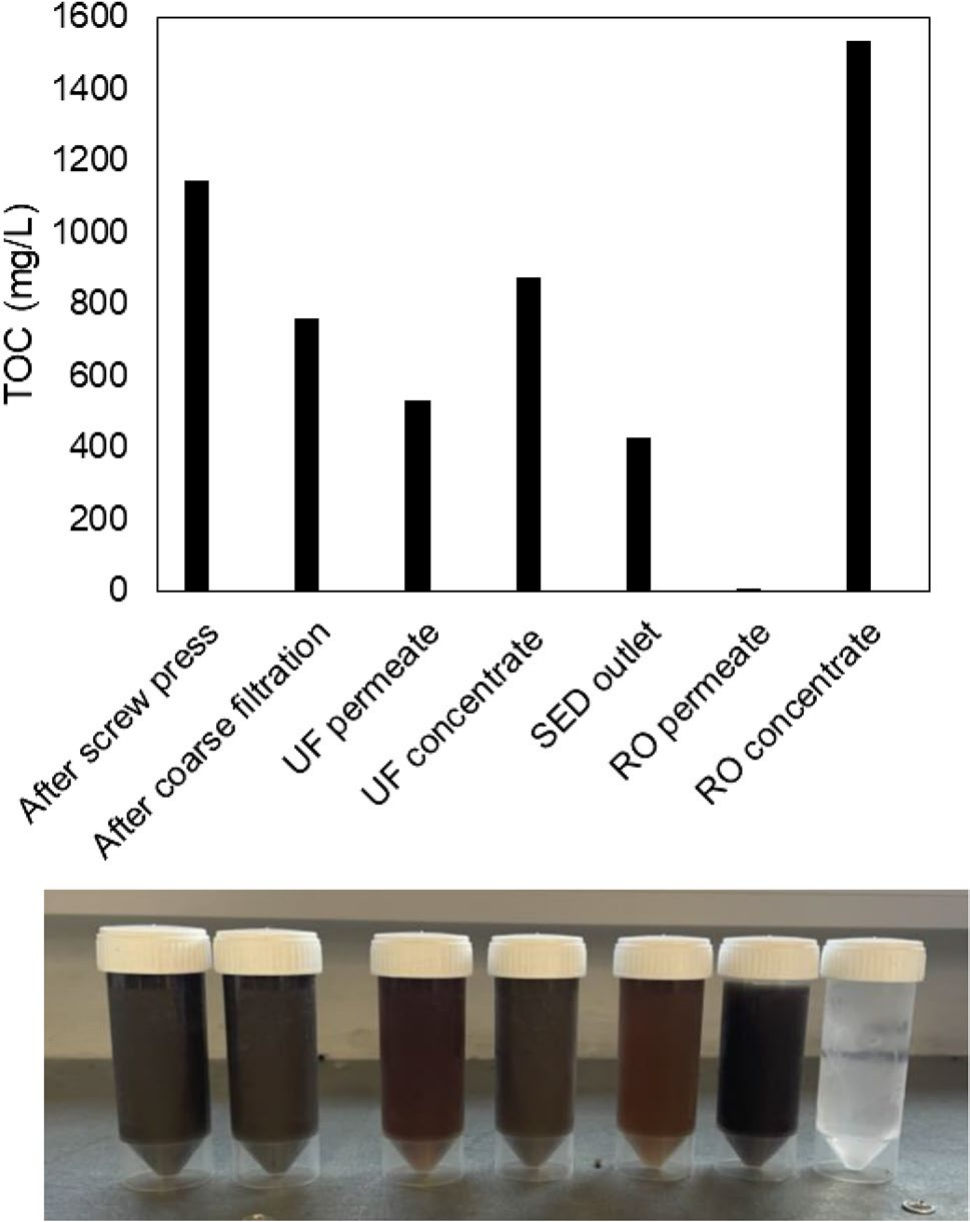
Total organic carbon (top) concentrations along the integrated membrane treatment process. The visible effect of these processes on the digestate color (bottom), from left to right: as received digestate, after coarse filtration, UF permeate, UF concentrate, SED outlet/RO feed, RO concentrate, RO permeate

In terms of TOC values, the top graph of Fig. 3 depicts a trend similar to that of the total solids. An initial concentration of about 1.20 g/L was reduced to 0.70 g/L by the initial application of coarse filtration, while the subsequent UF application led to a further 30% reduction in TOC in the permeate. This suggests that most of the particulate organics in the digestate are in particles larger than 1 µm and can be effectively retained by the MF process. During the removal of ions by the subsequent application of SED, the organic load was further reduced by approximately 20%, likely due to the adsorption of organics onto the SED membranes and the transfer of organic particles to the product streams. Overall, the treatment resulted in a permeate fraction with a TOC content of 1.50 mg/L, representing a total TOC reduction of about 99.8%. The produced RO concentrate presents an organic load 10 times higher than the permeate. The resulting permeate constituted approximately 83% of the volume treated by RO, indicating a relatively high water recovery during this treatment stage.

The bottom panel in Fig. 3 depicts the visible effect on the color of samples from different digestate fractions throughout the membrane processes application and demonstrates the impact of the integrated treatment process on the opacity of the produced solutions. The difference before and after coarse filtration is barely noticeable. However, a much more significant drop in opacity can be observed in the UF permeate, where the concentrate appears almost as opaque as the initial digestate. Additionally, there is a visible reduction in opacity between the UF permeate and the SED outlet, while the RO permeate appears completely clear. Finally, although the RO concentrate is very dark, it differs significantly from the digestate and the UF concentrate, most likely due to its very low suspended solids concentration.

### Nutrient flow

#### Nitrogen, potassium, and phosphorus

**Fig. 4:**
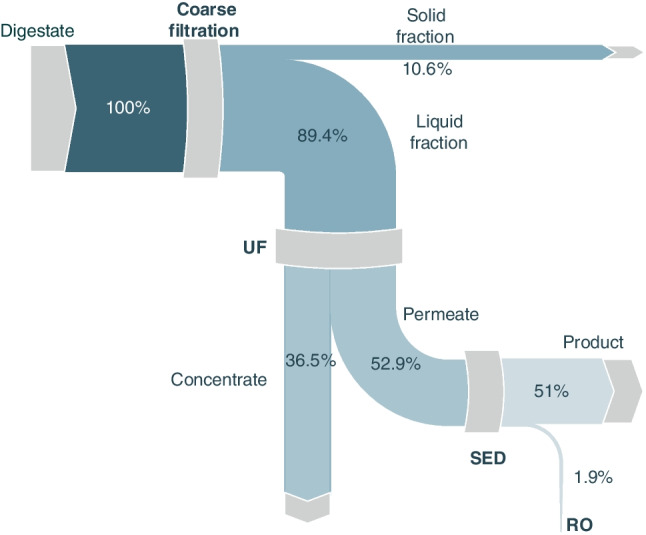
Ammonium fate through the integrated membrane processes of digestate

As aforementioned, the raw pig manure digestate used in this study contains relatively high concentrations of NH_4_^+^ (Table 1). The presence of ammonia concentration was monitored throughout the entire treatment process, and the respective results are illustrated in Fig. 4. Only 10.6% of NH_4_^+^ is separated with the solid fraction during the coarse filtration (initial) sub-process, while the remaining 89.4% is fed into the UF process. During ultrafiltration, a considerable amount of NH_4_^+^ is directed to the concentrate fraction, resulting in a secondary stream with a relatively high nitrogen content (1783.80 mg/L). However, the UF permeate contains 52.9% of the initial NH_4_^+^ found in the digestate, which is subsequently treated with SED. The SED product/output contains 51% of the feed NH_4_^+^, while the remaining 1.9% ends up in the RO concentrate.

**Fig. 5:**
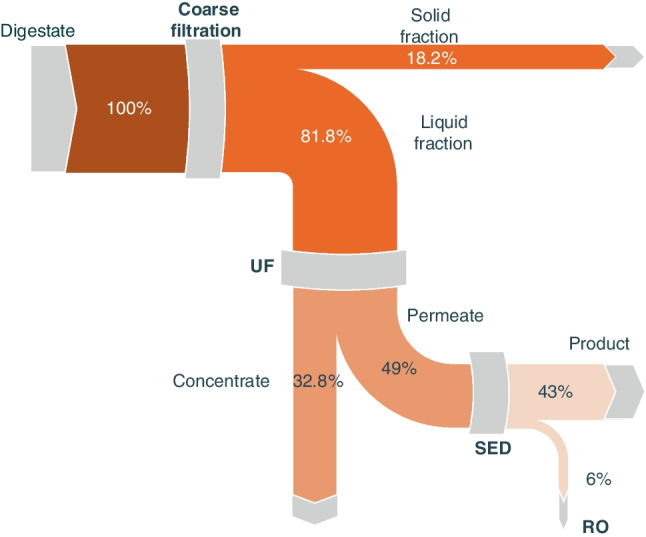
Potassium ion mass flow through the application of integrated membrane processes for the treatment of manure digestate

Potassium is considered indispensable for plant life, and it can be found in digestates in relatively high amounts, ranging from 1300.00 to 5200.00 mg/L (Drosg et al. 2015; WRAP 2012). Regarding commercially produced potassium compounds, almost 85% of them are used in agriculture as fertilizer, typically in the forms of potassium magnesium sulfate or potassium sulfate (U.S. Geological Survey 2020). In this study, the concentration of K^+^ in the treated digestate measured up to 1600.00 mg/L, nearly as high as the NH_4_^+^ concentration. Therefore, it becomes crucial to examine its fate and occurrence through the applied membrane treatment processes. The coarse filtration step did not show any significant influence on the K^+^ concentration in the liquid fraction, as only approximately 18% of its content followed the solid fraction. However, in the case of UF, a higher amount of K^+^ was found in the UF concentrate (40% of the initial K^+^), which is about twice of the corresponding amount noticed for the case of NH_4_^+^ (Fig. 5).

**Fig. 6:**
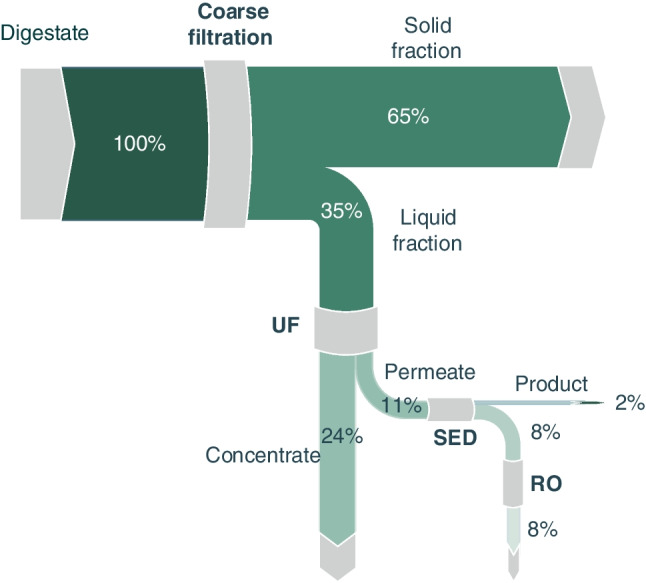
Phosphates fate through the application of integrated membrane processes for the treatment of manure digestate

Phosphorus is arguably the most important nutrient in digestates, at least from the economical perspective. It is one of the macro-nutrients that plants predominantly require for growth and typically obtained through the mining of respective mineral salts, often located in geographically limited areas (Daramola and Hatzell 2023). Hence, finding technoeconomically viable ways to recycle phosphorus from waste is considered particularly crucial. Phosphates, due to their chemical nature, are present in different fractions of the examined processes. After the coarse filtration step, approximately 65% of them resulted in the solid fraction, while only 11% remained in the UF permeate. As mentioned by other researchers (Al Seadi and Lukehurst 2012), phosphates tend to be mostly adsorbed onto solid particles, thus most of them end up in the solid streams/fractions (Fig. 6).

The selective electrodialysis (SED) process is subsequently employed to separate the ions from the pre-treated digestate and retrieve important plant nutrients in more concentrated product streams. Multiple experimental runs were conducted using the same product concentrate solutions but with different feed batches to maximize the concentration of nutrients. In all these runs, the voltage application was terminated when the conductivity of digestate reached 0.8 mS/cm, except for the last run, which was allowed to reach lower value (0.2 mS/cm).

**Fig. 7:**
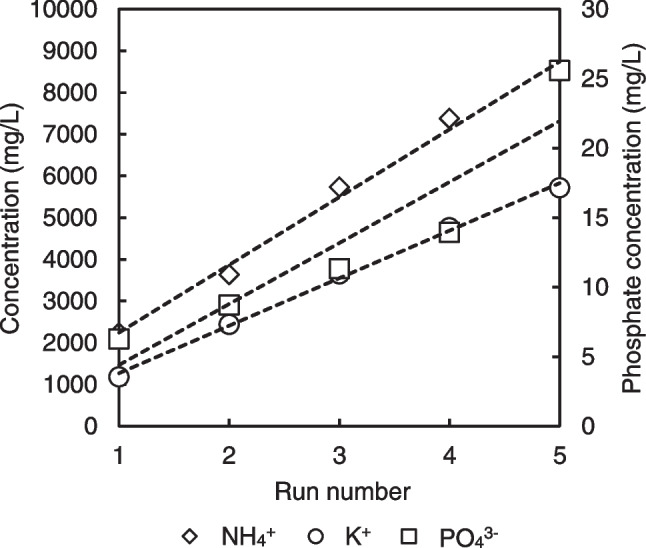
Concentrations of NH_4_^+^, K^+^, and phosphates in each product tank, after each of 5 consecutive experimental runs using different feed batches, but the same product collection tanks

Figure 7 shows the concentrations of NH_4_^+^ and K^+^ in tank C at the end of each run, as well as the concentration of phosphates in tank B. The separation process was highly effective for NH_4_^+^, achieving a separation of 96.4% of the feed ammonium in each run. Consequently, SED was able to retrieve just over half of digestate nitrogen (51%), as shown in Fig. 4.

Regarding the separation of K^+^ via SED, it exhibits similar behavior to NH_4_^+^, consistent with relevant literature (Li et al. 2022), achieving a high separation of approximately 95% compared to the feed concentration, with a near-constant concentration increment in the product tank after each run.

However, the concentration of phosphates in the SED feedstock was only 39.00 mg/L, and contrary to previous works conducted under relatively similar conditions (Ye et al. 2019), their retrieval was quite poor in this case. The average separation percentage was 20%, which may be explained by the larger size of phosphate anions and their consequent slower movement, as well as the high concentration of organics in the digestate, inhibiting the separation procedure. Organic molecules have been reported to primarily foul the anionic membranes, blocking the path of phosphates to the surface or through the membrane, thus resulting in low separation rates (Gurreri et al. 2020). A third factor likely contributing also to the lower separation rate of phosphates is their higher valence number.

Finally, as shown in Figs. 4, 5, and 6, after the SED treatment only 1.9% of the initial NH_4_^+^ remained in the RO feed (Fig. 4), with almost none passing through the membrane, thus the water produced can be practically described as free of nitrogen. In the case of K^+^ (Fig. 5), the RO permeate water contains only 0.66 mg K^+^/L. However, regarding phosphorus, the inefficiency of SED in recovering PO_4_^3−^ led to a higher concentration in the RO feedstock compared to the other ions. Nevertheless, phosphate-free water was produced in the latter unit, with the respective concentrate containing approximately 8% of the initial phosphate content (Fig. 6).

### Presence of heavy metals

Several metals, in the form of respective salts, such as Zn, Cr, Cd, Pb, Cu, and As, are commonly used as animal feed additives. Consequently, the produced animal manure may contain increased concentrations of these metals (Cang et al. 2004; McCarthy et al. 2013; Zhang et al. 2012). Relevant studies have shown that the content of these metals is not particularly affected during the anaerobic digestion (Lv et al. 2016). Therefore, the uncontrolled use of animal manure digestate as an agricultural fertilizer could lead to their gradual accumulation in the soil and potential contamination of agricultural plants (Buelna et al. 2008; Qureshi et al. 2008; Zhen et al. 2020).

In this study, the presence of Cd, Cr, Hg, Ni, Cu, Pb, Zn, and As was monitored throughout the membrane treatment process. Digestate samples after the preliminary screw press were found to contain only traces of the metals Cd, Cr, Hg, Ni, Pb, and As, at concentrations of 0.006, 0.880, 0.160, 1.140, 0.050, and 0.020 mg/L, respectively. Zn and Cu, due to their use as growth promoters, were found in higher concentrations, i.e., 84.100 mg/L and 4.020 mg/L, respectively.

**Fig. 8:**
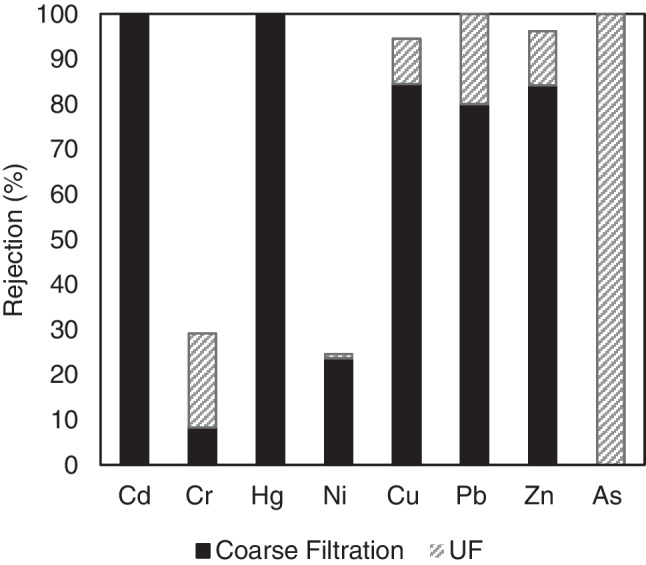
Heavy metals rejection rates after the application of MF and UF processes

Figure 8 depicts the rejection percentages through the application of coarse filtration and UF treatment. After the coarse filtration step, the presence of Cd and Hg was not detected in the liquid part of the digestate, whereas the Pb, Cu, and Zn content decreased by 80%, 85%, and 85%, respectively. The application of UF treatment resulted in the complete removal of As and Pb and almost complete removal for Cu and Zn. In contrast to the aforementioned metals, Ni and Cr rejection was relatively low, i.e., only 25% and 30%, respectively. Although these metals have been efficiently retained with the UF treatment in waters and wastewaters (presenting more than 90% rejection) (Lech et al. 2023; Muthumareeswaran et al. 2017), in this case the complexity of the digestate composition may have seriously affected their rejection rate by the membrane treatment processes.

The main initial filtration processes, i.e., the coarse filtration and the UF, resulted in a UF permeate fraction, containing 0.190 mg/L Cr, 0.410 mg/L Ni, 2.850 mg/L Zn, and 0.170 mg/L Cu. In all these cases, including the untreated digestate and the UF permeate, the heavy metal content falls below the respective regulation limits for the further fertilizer application of liquid fractions (The European Parliament and the Council of the European Union 2019). Therefore, heavy metals concentrations were not monitored in the fractions resulting after the UF treatment.

### Antibiotics

Several studies have reported the presence of considerable amounts of organic contaminants, such as antibiotics, in animal manure (Chan et al. 2020; Gros et al. 2019; Martínez-Carballo et al. 2007; Qiao et al. 2012; Spielmeyer 2018). A relevant screening study by a European research center, regarding conventional pig husbandry during 2011 and 2013, recorded 34 different antibiotics used in 21 farms and all of them were detectable in the resulting manure (Widyasari-Mehta et al. 2016). Furthermore, antibiotic residues in digestates indicate that anaerobic digestion cannot fully eliminate these mostly refractory, complex organic compounds (Feng et al. 2017). Lehmann and Bloem (2021) studied 29 biogas plants from three different countries and detected antibiotics in 83% of the examined digestates. Spielmeyer (2018) reported wide ranges of antibiotics removal during the anaerobic digestion treatment, ranging from 27 to 99% for oxytetracycline, 7 to 99% for chlortetracycline, 12 to 99% for tetracycline, and 0 to 95% for sulfamethazine, depending on both experimental setups and the concentration and type of solids. Thus, the application of untreated digestate as soil amender can potentially pose a serious threat for soil contamination with the gradual accumulation of both antibiotics and antibiotic resistance genes (ARGs), disturbing the soil microbial ecology (Gurmessa et al. 2020; Tasho and Cho. 2016).

**Fig. 9:**
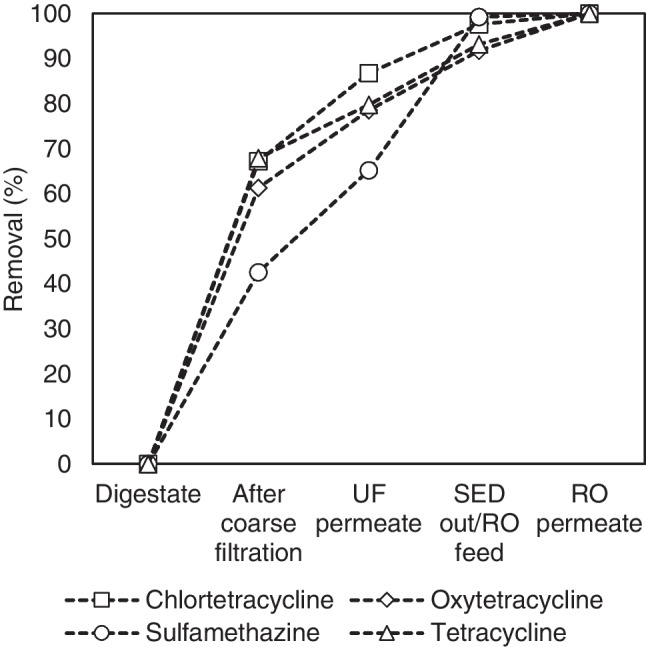
Antibiotics removal through the integrated membrane treatment processes of manure digestate

In this study, 60 antibiotics (the list is provided in Table S1 of the supplementary materials) were analyzed in samples of digestate, as well as throughout the overall membrane treatment processes. Only 4 antibiotics were detected, belonging to two main classes, i.e., chlortetracycline (CTC), oxytetracycline (OTC), tetracycline (TC), and sulfamethazine (SMT). Figure 9 depicts the removal of these antibiotics through the different process stages.

After the coarse filtration step, the presence of CTC, OTC, and TC dropped to 32–38%, while the SMT still remained at higher percentages (approximately 57%) compared to the initial concentration values of digestate. In the cases of CTC, OTC, and TC, their removal during UF treatment was approximately 30%, while in the case of SMT, it was 37%.

The SED treatment did not affect the presence of tetracycline-class antibiotics but eliminated the presence of SMT, indicating that it was either adsorbed onto the membrane or transferred to one of the SED products (see also Shi et al. 2020). The water produced after the RO treatment was antibiotic-free, while the RO concentrate contained less than 5% of the initial amount of antibiotics.

According to these findings, the application of membrane processes has been proved to be efficient in terms of producing antibiotic-free water. However, they do not have a specific mechanism to eliminate or destroy them, as this could be performed by the application, for example, of AOPs (Moradi et al. 2024), and the antibiotics end up in the concentrate streams or adsorbed onto the membranes themselves. More specifically, the UF concentrate contained 15.40 µg/L of CTC and OTC, 3.20 µg/L of TC, and 42.20 µg/L of SMT, while the RO concentrate 2.00 µg/L of CTC, 12.50 µg/L of OTC, 1.40 µg/L of TC, and 34.40 µg/L of SMT.

### Potential products from the integrated membrane treatment processes

#### Water

Water scarcity in the EU is becoming an increasingly important problem, especially in the southern countries. Reusing reclaimed water (after proper treatment) is a generally accepted practice that supports the EU’s overall plan, as outlined in the European Green Deal, by improving water resource management and assisting the adaptation of water systems to climate change. The goal of Regulation EU Dir. 2020/741 of the European Parliament and of the Council on Minimum Requirements for Water Reuse (known as the Water Reuse Regulation) is to make the EU food system more sustainable and resilient while safeguarding both of these important aspects, i.e., public health and the environment. Agriculture is a sector that can be particularly vulnerable to scarce or intermittent water resources. Effective from 26 June 2023, the Water Reuse Regulation establishes universal minimum water quality requirements for the safe reuse of treated urban waste water in agricultural irrigation.

In this study, the digestate consisted of approximately 98% water, and it was possible to reclaim 50.7% of this at the end of the integrated membrane treatment processes as the RO permeate stream. The water produced was found to be of good quality, without color and almost completely deionized. Table 2 presents the minimum quality requirements for water reuse in agricultural irrigation. The results of TSS and turbidity were 8.00 mg/L and 3.00 NTU, respectively, complying with legislative limits. However, in the case of BOD_5_, the RO permeate exceeded the stringent minimum requirements (34.00 mg/L). Furthermore, the microbial burden of reclaimed water should also be examined. Nevertheless, agricultural water reuse may be feasible in other sectors (e.g., for cotton, trees, etc.), where the respective quality limits are less strict.

**Table 2:** Reclaimed water quality requirements for agricultural irrigation

Reclaimed water quality class	*Escherichia coli* (number/100 mL)	BOD_5_ (mg/L)	TSS (mg/L)	Turbidity (NTU)
All food crops consumed raw where the edible part is in direct contact with reclaimed water and root crops consumed raw	≤ 10	≤ 10	≤ 10	≤ 5
Water recovered in this study	–	34	8	3

#### SED products

SED can be considered as an electrically driven process that extracts nutrient ions from the feed solution to the product solutions, as previously described in Proskynitopoulou et al. (2022). In contrast to other membrane treatment processes, SED has the main advantage of concentrating and selectively separating nutrients into different product solutions. Thus, it is possible to mix the products, i.e., cationic and anionic constituents, and to precipitate or create valuable products, such as alternative fertilizers for potential agricultural use, replacing those chemically produced (Ye et al. 2019).

SED achieves high ammonium recovery with the brine product, as shown in Fig. 4. After multiple runs, ammonium concentration can be 4.6 times higher, than the initial digestate treated, making the product a rich nitrogen source. However, direct application of this product as fertilizer is not feasible due to the relatively high of NaCl concentrations (around 10 g/L). Hence, further separation of the nitrogen is necessary. Ammonia stripping, a well-known separation procedure, could be employed on the brine product to achieve final ammonium recovery as ammonium sulfate fertilizer (Li et al. 2022).

The brine product also contains high concentrations of potassium, reaching up to 5721.80 mg/L. However, recovering potassium as a reusable fertilizer salt could be quite challenging. Nevertheless, mixing the anionic (mainly phosphates-rich), cationic (mainly magnesium-rich), and brine (mainly potassium-rich) separated streams could result in the MgKPO_4_∙6H_2_O (MPP) product, considered as a slow release fertilizer (Larsen et al. 2013). However, relevant studies have shown that the presence of ammonium can also favor the precipitation of MgNH_4_PO_4_ product (MAP) over the MPP (Johansson et al. 2019; Xu et al. 2015). Another study recovered approximately 70% of potassium as potassium bitartrate from the respective wastewaters by using tartaric acid, and subsequently, converting to KNO_3_ using Mg(OH)_2_ and HNO_3_ (Khatri and Garg 2022).

Phosphorus could be recovered via selective precipitation from the anionic product, e.g., as salts of different compositions, such as NH_4_MgPO_4_, KMgPO_4_, Ca_3_(PO_4_)_2_, Mg_3_(PO_4_)_2_, NH_4_H_2_PO_4_, or (NH_4_)_2_HPO_4_ (Ye et al. 2019). In this study, the potential phosphorus salts precipitation was examined. For this purpose, MINEQL + (a specific chemical equilibrium calculations software) was used. The results showed that by adding calcium at the stoichiometric molar ratio of Ca/P = 5/3 and at pH 9.5, 99.2% of phosphorus could be precipitated in the form of hydroxyapatite (Ca_10_(PO_4_)_6_(OH)_2_). In the case of MAP precipitation, only ~ 70% of phosphates from the anionic product could be recovered, and this was after the addition of substantially more magnesium (15 times higher than the respective stoichiometric molar ratio, Mg/P 1/1).

#### Concentrates

Concentrates were produced during the treatment of different digestate fractions using pressure-driven membrane processes. The UF concentrate consists of 33% of the initial digestate volume. This nutrient-rich fraction contains approximately 35% of the digestate’s available ammonium and potassium content and approximately 24% of its phosphorus content. Additionally, 32.8% of digestate total solids end up in this concentrate fraction, along with 75% of TOC. It is worth noting that the heavy metals content is below the legislation limits for application as agricultural fertilizer (The European Parliament and the Council of the European Union 2019).

The RO concentrate consists of only 6.3% of the initial digestate volume treated. This fraction contains only a small part of the initial ammonium and potassium, i.e., 1.9% and 6%, respectively. On the contrary, the phosphorus content (8%) was considerable in relation to the UF permeate, which contained only 11% of digestate phosphorus. The high concentration of phosphorus in the RO concentrate is attributed to the poor SED separation efficiency for phosphates, as aforementioned. Approximately 1% of digestate solids reach this fraction, while the organic concentration was 1.3 times higher, than in the untreated digestate.

The presence of antibiotics in the concentrates of UF and RO processes is a limiting factor for the field application of these streams. Further treatment with other technologies should be investigated, e.g., by selective oxidation or adsorption, aiming to efficiently eliminate these organic compounds (Yang et al. 2022).

## Conclusions

Pig manure digestate was successfully treated by the application of different membrane processes applied in series. A preliminary coarse filtration process, involving sieving and microfiltration, was found to efficiently remove solids larger than 1 µm, while a subsequent ultrafiltration process can remove solids and organic molecules larger than 20 nm. The inorganic nutrients (NH_4_^+^, K^+^) were extracted from the filtered digestate by the application of selective electrodialysis at rates above 90%, except for the case of phosphates (~ 20%). The digestate was then fed to a reverse osmosis unit, where almost all the remaining nutrients and most of the organics content were retained with the concentrate stream, while the permeate (considered as clean water) constituted just over half of the digestate volume treated. Analytical determinations of antibiotics showed that they tended to follow the solids rather than the dissolved phase, with 40–65% of all examined types being retained by the coarse filtration combined step and a further ~ 20% by the ultrafiltration unit, while about a quarter of all sulfamethazines were retained in the electrodialysis unit. A similar trend can be observed for the presence of heavy metals, with most of them being completely eliminated in the UF permeate.

The water recovered by reverse osmosis was within the reuse limits of the EU in terms of nutrients, suspended solids, and turbidity, and only slightly above in terms of BOD_5_ content. The UF and RO concentrates are of particular interest as potential soil amenders, but their antibiotics content must be specifically addressed, while the presence of heavy metals in the soil must also be monitored over time. Finally, the SED concentrates can be further processed for the production of fertilizers tailored to farmers’ soil and crop requirements, aiming to replace conventional, non-renewable mineral fertilizers.

## Supplementary Information

Below is the link to the electronic supplementary material.

Supplementary file1 (PDF 180 KB)
