# Supplementary material for: Treatment of anaerobically digested pig manure by applying membrane processes for nutrient recovery and antibiotics removal
Source: Environ Sci Pollut Res Int. 2024 Apr 13;32(48):27715–27. doi: 10.1007/s11356-024-33313-x (PMC12696056; doi:10.1007/s11356-024-33313-x)
Supplement: Supplementary file 1 — Supplementary file1 (PDF 180 KB) [file 11356_2024_33313_MOESM1_ESM.pdf]

Treatment of anaerobically digested pig manure by applying membrane processes for nutrient recovery and antibiotics removal

Vera Proskynitopoulou [1,2*], Anastasios Vourros [1], Ioannis Garagounis [1], Panagiotis Dimopoulos Toursidis [1], Souzana Lorentzou [1], Panagiotis Kougias [3], Anastasios Zouboulis [2] and Kyriakos Panopoulos [1]

1 ARTEMIS Laboratory, Chemical Process and Energy Resources Institute, Centre for Research & Technology Hellas, 57001 Thessaloniki, Greece
2 Chemical and Environmental Technology Laboratory, Department of Chemistry, Aristotle University of Thessaloniki, 54124 Thessaloniki, Greece
3 Hellenic Agricultural Organisation-DEMETER, Soil and Water Resources Institute, 57001 Thessaloniki, Greece

* Correspondence: verapros@certh.gr

Table S1. List of antibiotics analyzed in different process stages.

**Amoxicillin**
**Azithromycin**
**Chlortetracycline**
**Ciprofloxacin**
**Clarithromycin**
**Danofloxacin**
**Dexamethasone**
**Difloxacin**
**Doxycycline**
**Enoxacin**
**Enrofloxacin**
**Erythromycin**
**Fenbendazole**
**Fleroxacin**

**Flumequine**
**Lincomycin**
**Lomefloxacin**
**Marbofloxacin**
**Metronidazole**
**Nalidixic acid**
**Norfloxacin**
**Ofloxacin**
**Ormetoprim**
**Oxacillin**
**Oxolinic acid**
**Oxytetracycline**
**Pefloxacine***
**Penicillin G**
**Progesterone**
**Roxithromycin**
**Sarafloxacin**
**Sparfloxacin**
**Sulfabenzamide**
**Sulfacetamide**
**Sulfachlorpyridazine**
**Sulfaclozine**

**Sulfadiazine**
**Sulfadimethoxine**
**Sulfadoxine**
**Sulfaguanidine**
**Sulfamerazine**
**Sulfameter**
**Sulfamethazine**
**Sulfamethizole**
**Sulfamethoxazole**
**Sulfamethoxypyridazine**
**Sulfamonomethoxine**
**Sulfamoxole**
**Sulfanilamide**
**Sulfapyridine**
**Sulfaquinoxaline**
**Sulfathiazole**
**Sulfisomidine**
**Sulfisoxazole**
**Tetracycline**
**Tiamulin**
**Tilmicosin**

**Trimethoprim**
**Tylosin**
**Valnemulin**
